# Influenza vaccination and myocarditis among patients receiving immune checkpoint inhibitors

**DOI:** 10.1186/s40425-019-0535-y

**Published:** 2019-02-22

**Authors:** Magid Awadalla, Doll Lauren Alexandra Golden, Syed S. Mahmood, Raza M. Alvi, Nathaniel D. Mercaldo, Malek Z. O. Hassan, Dahlia Banerji, Adam Rokicki, Connor Mulligan, Sean P. T. Murphy, Maeve Jones-O’Connor, Justine V. Cohen, Lucie M. Heinzerling, Merna Armanious, Ryan J. Sullivan, Rongras Damrongwatanasuk, Carol L. Chen, Dipti Gupta, Michael C. Kirchberger, Javid J. Moslehi, Sachin P. Shah, Sarju Ganatra, Paaladinesh Thavendiranathan, Muhammad A. Rizvi, Gagan Sahni, Alexander R. Lyon, Carlo G. Tocchetti, Valentina Mercurio, Franck Thuny, Stephane Ederhy, Michael Mahmoudi, Donald P. Lawrence, John D. Groarke, Anju Nohria, Michael G. Fradley, Kerry L. Reynolds, Tomas G. Neilan

**Affiliations:** 1 Cardiac MR PET CT Program, Department of Radiology, Massachusetts General Hospital, 165 Cambridge Street, Suite 400, Boston, MA 02114 USA; 2 Cardiology Division, New York-Presbyterian Hospital, Weill Cornell Medical Center, New York, NY USA; 3 Division of Oncology and Hematology, Department of Medicine, Massachusetts General Hospital, Boston, MA USA; 4 Department of Dermatology, University Hospital Erlangen, Friedrich-Alexander-University Erlangen-Nurnberg (FAU), Erlangen, Germany; 5 Cardio-Oncology Program, H. Lee Moffitt Cancer Center & Research Institute and University of South Florida Division of Cardiovascular Medicine, Tampa, FL USA; 6 Cardiology Division, Memorial Sloan Kettering Cancer Center, Weill Cornell Medical College, New York, NY USA; 7 Cardio-Oncology Program, Vanderbilt University Medical Center, Nashville, TN USA; 8 Cardiology Division, Lahey Hospital & Medical Center, Burlington, MA USA; 9 Ted Rogers Program in Cardiotoxicity Prevention, Peter Munk Cardiac Center, Division of Cardiology Toronto General Hospital, University of Toronto, Toronto, Ontario Canada; 10 Division of Oncology and Hematology, Department of Medicine, Lehigh Valley Hospital, Allentown, PA USA; 11 The Mount Sinai Hospital, New York, NY USA; 12 Cardio-Oncology Program, Royal Brompton Hospital, London, UK; 13 Department of Translational Medical Sciences, Federico II University, Naples, Italy; 14 Cardiovascular Division, Department of Medicine, Aix-Marseille Universite, Marseille, France; 15 Cardio-Oncology Program, Division of Cardiology, Hopitaux Universitaires est Paris, Paris, France; 16 Division of Cardiology, Department of Medicine, Southampton General Hospital, Southampton, UK; 17 Cardio-Oncology Program, Division of Cardiology, Department of Medicine, Brigham and Women’s Hospital, Boston, MA USA; 18 Cardio-Oncology Program, Division of Cardiology, Department of Medicine, Massachusetts General Hospital, Boston, MA USA

**Keywords:** Influenza vaccination, Immune checkpoint inhibitors, Myocarditis, Cancer, Immune-related adverse events, Major adverse cardiac events, Cardiovascular disease

## Abstract

**Background:**

Influenza vaccination (FV) is recommended for patients with cancer. Recent data suggested that the administration of the FV was associated with an increase in immune-related adverse events (irAEs) among patients on immune checkpoint inhibitors (ICIs). Myocarditis is an uncommon but serious complication of ICIs and may also result from infection with influenza. There are no data testing the relationship between FV and the development of myocarditis on ICIs.

**Methods:**

Patients on ICIs who developed myocarditis (*n* = 101) (cases) were compared to ICI-treated patients (*n* = 201) without myocarditis (controls). A patient was defined as having the FV if they were administered the FV from 6 months prior to start of ICI to anytime during ICI therapy. Alternate thresholds for FV status were also tested. The primary comparison of interest was the rate of FV between cases and controls. Patients with myocarditis were followed for major adverse cardiac events (MACE), defined as the composite of cardiogenic shock, cardiac arrest, hemodynamically significant complete heart block and cardiovascular death.

**Results:**

The FV was administered to 25% of the myocarditis cases compared to 40% of the non-myocarditis ICI-treated controls (*p* = 0.01). Similar findings of lower rates of FV administration were noted among myocarditis cases when alternate thresholds were tested. Among the myocarditis cases, those who were vaccinated had 3-fold lower troponin levels when compared to unvaccinated cases (FV vs. No FV: 0.12 [0.02, 0.47] vs. 0.40 [0.11, 1.26] ng/ml, *p* = 0.02). Within myocarditis cases, those administered the FV also had a lower rate of other irAEs when compared to unvaccinated cases (36 vs. 55% *p* = 0.10) including lower rates of pneumonitis (12 vs. 36%, *p* = 0.03). During follow-up (175 [IQR 89, 363] days), 47% of myocarditis cases experienced a MACE. Myocarditis cases who received the FV were at a lower risk of cumulative MACE when compared to unvaccinated cases (24 vs. 59%, *p* = 0.002).

**Conclusion:**

The rate of FV among ICI-related myocarditis cases was lower than controls on ICIs who did not develop myocarditis. In those who developed myocarditis related to an ICI, there was less myocardial injury and a lower risk of MACE among those who were administered the FV.

**Electronic supplementary material:**

The online version of this article (10.1186/s40425-019-0535-y) contains supplementary material, which is available to authorized users.

## Introduction

Immune checkpoint inhibitors (ICIs) have revolutionized the care of several groups of patients with advanced cancers [1]. These therapies are approved for use among patients with metastatic melanoma, non-small cell lung cancer, squamous carcinoma of the head and neck, renal cell carcinoma, Hodgkin’s lymphoma, liver cancer, squamous cell of the skin and bladder cancer [2–8]. Checkpoint inhibitors are predominantly approved in late stage patients but, due to efficacy, are being expanded to adjuvant settings [9–13]. They work by releasing negative regulators of immune activation, thus facilitating the recognition of tumors by the immune system. As anticipated, activation of the immune system may result in immune-mediated adverse effects (irAEs) [14]. Myocarditis is an uncommon but serious immune complication of ICIs [15–22], myocarditis related to an ICI occurs early after initiation of ICIs [15] and the development of myocarditis after ICIs is associated with significant morbidity and mortality [15, 20–24]. The risk factors for the development of myocarditis among patients on ICI therapy are poorly understood [16, 25] and an improved understanding is needed. In this submission, we tested the effect of administration of the FV on the development of myocarditis. By way of background, there is controversy as to whether it is safe to administer the influenza vaccine to patients receiving immunotherapy and there are data in patients at risk of cardiovascular disease that the influenza vaccine may be protective. The national comprehensive cancer network (NCCN) guidelines recommend the FV in patients with hematologic or solid tumor malignancies, but there are no guidelines specific to those on immune therapy [26]. In a recent study, with 23 lung cancer patients on ICI (cases) and 11 age-matched healthy controls, influenza vaccination was associated with a heightened immune and inflammatory response resulting in a high rate of general irAEs (52.2%) [27]. In contrast, in a multi-center study with a broad range of cancers, FV administration was associated with a non-significant increase in overall survival among patients on ICI’s [28] and in a single center retrospective study of over 500 patients, a similar rate of general irAEs were noted between vaccinated (37.4%) and unvaccinated patients (42.6%) [29]. However, the majority of the irAEs in that study (87%) were ICI- related pneumonitis and there are no studies to date testing the association of the FV and development of ICI related myocarditis [29]. Therefore, the goal of this study is to test the association between FV and the development of myocarditis among patients on ICI’s. This relationship between FV status and the development of myocarditis may be of additional importance as the development of influenza infection is also, albeit rarely, associated itself with an increased risk for myocarditis and major adverse cardiovascular events [30–32]. Additional goals included testing the effect of FV status on outcomes among those patients who develop myocarditis.

## Methods

### Patients

Cases were derived from a 16-center institutional registry, which was created to collate cases of ICI-related myocarditis. The cases were diagnosed between November 2013 and October 2018. Controls were derived from a single-center registry (Massachusetts General Hospital, Boston, Massachusetts) of all patients started on ICI in the same time interval who did not develop myocarditis. The number of patients treated with ICI therapy at Massachusetts General Hospital during the study period was confirmed by 2 independent researchers. Controls, in a 2:1 ratio, were randomly selected and not pre-selected to match cases on any variables. The study was approved by each center’s institutional review board, and the requirement for written informed consent was waived.

### Covariates

Data on covariates of interest were retrospectively extracted from electronic medical records and included standard demographics, cardiovascular risk factors, medication, and echocardiographic variables. Cancer-specific covariates included the cancer type, ICI treatment, prior cardiotoxic chemotherapy, and prior radiation therapy. Myocarditis specific covariates included clinical presentation, physical examination, cardiac biomarkers, and echocardiographic parameters.

### Definitions and outcome of interest

The diagnosis of myocarditis was made by one of two standard methods; 1. The presence of standard histological features present on endomyocardial biopsy or autopsy or 2. A guideline-recommended standardized scoring system which incorporates clinical, biomarker and cardiac imaging features [33]. Subjects were defined as having received the FV if they received the FV anytime from 6 months prior to starting ICI to receiving the FV while on ICI therapy. This time frame was chosen as numerous studies have shown the period of effectiveness of the vaccine ranges within different cohorts, but peaks at 4–6 months, after which significantly declines [34, 35]. The administration of FV was at the discretion of clinician involved in care and not performed as part of a study. Two alternate thresholds to define FV status were also tested. In a second analysis, we defined FV status based on receiving the FV anytime from 3 months prior to starting ICI to receiving the FV while on ICI therapy. In the third definition of FV status, the FV group was restricted to those who were administered the FV after starting on an ICI. The first comparison was between cases who developed myocarditis and controls who did not develop myocarditis, separated by FV status. Additional analyses performed were restricted only to myocarditis cases. Within the cases who developed myocarditis, we next tested the association between FV status and adverse cardiovascular outcomes after the development of myocarditis. Major adverse cardiac events (MACE) was defined, as per prior studies among patients on ICI, as a composite of cardiovascular death, cardiac arrest, cardiogenic shock, and hemodynamically significant complete heart block (CHB) [15]. In cases where cardiac arrest, cardiogenic shock, or CHB led to death, that case was counted as a cardiac death. Standard definitions were used for cardiovascular death [36], cardiac arrest [37], and cardiogenic shock [38]. Hemodynamically significant CHB was defined as a complete absence of atrial-to-ventricular conduction requiring a temporary pacemaker [39].

### Statistical analysis

Continuous variables were summarized as either the mean ± standard deviation (SD) or as the median and interquartile range (IQR), as appropriate, and categorical variables were presented as percentages. Comparisons by case status (case vs. control) and by flu vaccination status were compared using the Student’s *t*-test for continuous variables or either the chi-square or Fisher’s exact test for categorical variables. Kaplan Meier curves and the log-rank test were generated to quantify the relationship between FV and MACE-free survival. All statistical tests were 2-sided and 5% was set as the level of significance. Statistical analysis was performed using R Version 3.5.1 (R foundation for statistical computing, Vienna, Austria).

## Results

### Patient characteristics

The mean age of patients (*n* = 101) who developed ICI-associated myocarditis was 67 ± 18 years with 72% being male (Table 1). The median time to onset of myocarditis from first ICI was 57 days (interquartile range 27–122 days). In comparison with controls (*n* = 201), myocarditis cases had a higher body mass index (Table 1); otherwise, there were no major differences in non-cancer variables between cases and controls. The most common presentations were chest pain and shortness of breath (Table 3). An echocardiogram was performed in 98% (99/101) of cases; 41% (41/99) had a reduced ejection fraction (EF) (< 50%) and 59% had a preserved EF.

**Table 1:** Description of cases and controls

	Myocarditis (*n* = 101)	Controls (*n* = 201)	*P Value*
Age at start of ICI, yrs	67 ± 18	64 ± 14	0.15
Male	73 (72)	129 (64)	0.16
*CV risk factors*			
Current or prior smoking	40 (47)	110 (58)	0.10
Hypertension	59 (60)	115 (61)	0.88
Diabetes mellitus	22 (23)	29 (15)	0.09
No CV risk factors	23 (23)	40 (20)	0.56
Coronary artery disease	12 (13)	24 (13)	0.86
Stroke	7 (8)	22 (12)	0.32
Heart failure	5 (6)	13 (7)	0.69
COPD	12 (14)	25 (13)	0.87
Obstructive sleep apnea	6 (7)	11 (6)	0.70
Chronic kidney disease^a^	9 (11)	31 (16)	0.22
Body mass index, kg/m^2^	28 ± 7	26 ± 6	0.01
*Primary cancer type*			
Head and neck	5 (5)	14 (7)	0.50
Hodgkin’s lymphoma	2 (2)	2 (1)	0.60
Melanoma	44 (44)	100 (50)	0.31
Lung cancer	17 (17)	35 (17)	1.00
Pancreatic	2 (2)	0	0.11
Renal cell carcinoma	6 (6)	3 (1)	0.07
Glioblastoma	2 (2)	2 (1)	0.60
Other	23 (23)	20 (10)	0.005
*Prior chemotherapy or radiation*			
Radiation	29 (29)	108 (54)	< 0.001
Anthracyclines	6 (6)	3 (1)	0.07
Cyclophosphamide	2 (2)	2 (1)	0.60
Gemcitabine	5 (5)	8 (4)	0.77
Taxanes	6 (6)	32 (16)	0.01
Carboplatin	8 (8)	60 (30)	< 0.001
VEGF Inhibitors	1 (1)	7 (3)	0.28
*Pre-ICI home CV medications*			
Statin	32 (37)	45 (24)	0.02
Aspirin	23 (26)	42 (22)	0.43
Beta-blockers	24 (28)	55 (29)	0.84
ACE inhibitors or ARB	26 (30)	38 (20)	0.07
Calcium-channel blocker	8 (9)	33 (17)	0.08
*Rate of influenza vaccination*			
6 months:			
6 months prior to ICI or on ICI	25 (25)	80 (40)	0.01
Time of vaccination prior to ICI, days	88 [25, 120]	79 [43, 170]	0.53
3 months:			
3 months prior to ICI or on ICI	17 (17)	69 (34)	0.002
Time of vaccination prior to ICI, days	31 [6,85]	44 [13,58]	0.88
On ICI therapy only:			
On ICI	8 (8)	34 (17)	0.04

Values are mean ± SD or n (%), unless otherwise indicated. ^a^Chronic kidney disease = glomerular filtration rate < 60 ml/min/1.73 m2. *ICI* immune checkpoint inhibitors, *CV* cardiovascular, *COPD* chronic obstructive pulmonary disease, *VEGF* vascular endothelial growth factor, *ACE* angiotensin converting enzyme, *ARB* angiotensin receptor blockers

### Cancer and treatment characteristics

The most common indications for ICI were melanoma and non-small cell lung cancer (Table 1). Compared to controls, the myocarditis cases were less likely to have had prior radiation therapy, taxol or carboplatin chemotherapy (Table 1). When compared to the control group without myocarditis, the myocarditis cases were also more likely to have received combination ICI therapy (Table 2). However, overall, most cases of myocarditis were being treated with concurrent single ICI therapy (72%). A complete description of the ICI therapies between cases and controls separated by those on combination therapy or single therapy at presentation is shown in Table 2. The median follow-up time was 290 [IQR 139,543] days for controls, and 175 [89,363] days for myocarditis cases (Table 2). 50% of the myocarditis cases had not experienced another ICI-related side effect. There was generally no difference in the overall prevalence of other ICI-related side effects between cases and controls; however, myocarditis cases who did have an additional previous immune-related side effect had higher rates of pneumonitis and neurological side effects (Table 2).

**Table 2:** Baseline cancer demographics

	Cases (*n* = 101)	Controls (*n* = 201)	*P* value
Single agent vs. combined			
Combination	28 (28)	14 (7)	< 0.001
Monotherapy	73 (72)	177 (93)	< 0.001
Combined ICI			
Ipilimumab (anti-CTLA4) + nivolumab (anti-PD1)	24 (24)	13 (6)	< 0.001
Ipilimumab (anti-CTLA4) + pembrolizumab (anti-PD1)	1 (1)	0	0.33
Tremelimumab (anti-CTLA4) + avelumab (anti-PD1)	1 (1)	0	0.33
Tremelimumab (anti-CTLA4) + durvalumab (anti-PD1)	2 (2)	1 (0)	0.26
Monotherapy ICI^a^			
Pembrolizumab (anti-PD1)	35 (35)	62 (31)	0.50
Nivolumab (anti-PD1)	25 (25)	85 (42)	0.003
Ipilimumab (anti-CTLA4)	6 (6)	28 (14)	0.04
Tremelimumab (anti-CTLA4)	1 (1)	0	0.33
Atezolizumab (anti-PDL1)	6 (6)	2 (1)	0.02
Avelumab (anti-PDL1)	0	0	1.00
Durvalumab (anti-PDL1)	0	0	1.00
Overall types of ICI			
Any anti-PD1	85 (84)	160 (80)	0.34
Any anti-CTLA4	35 (35)	42 (21)	0.01
Any anti-PDL1	9 (9)	3 (1)	0.003
Days of follow-up [IQR]	175 [89,363]	290 [139,543]	< 0.001
Other immune side effects during treatment^b^			
No other immune side effects	51 (50)	86 (43)	0.20
Hypophysitis/pituitary/adrenal	6 (6)	14 (7)	0.74
Pneumonitis	30 (30)	24 (12)	< 0.001
Hepatitis	8 (8)	11 (5)	0.41
Colitis	9 (9)	27 (13)	0.25
Dermatitis	6 (6)	5 (2)	0.19
Neurological	11 (11)	4 (2)	0.001
Gastritis	3 (3)	5 (2)	1.00

Values are n (%) or mean ± SD. All cases with ICI-associated myocarditis had ICI permanently discontinued. ^a^If most recent ICI therapy was monotherapy. ^b^More than one immune side effect may occur. *Anti-CTLA4* anti-cytotoxic T-lymphocyte-associated protein 4, *anti-PD1* anti-programmed cell death protein 1, *anti-PDL1* anti-programmed death-ligand 1, *ICI* immune checkpoint inhibitors

### Influenza vaccination

Within 6 months prior to starting or during ICI treatment, 25% (25/101) of the myocarditis cases received the FV (median of 88 days, interquartile range 25–120 days). In comparison, FV was administered to 40% (80/201, *p* = 0.01 for rate comparison) of controls on an ICI who did not develop myocarditis (median of 79 days, interquartile range of 43–170, Table 1). We also restricted the comparison of FV rates to cases from the institution where the controls were also derived (MGH). We found that in an analysis restricted to myocarditis cases at MGH, the rate of FV among cases was 17% (5/30, *p* = 0.02). Additional time-cut offs in the larger cohort were also tested to define whether a patient received the FV. In a second cut-off, we defined FV as having been administered the FV within 3 months prior to starting ICI treatment or during ICI therapy. When implementing this second time-cut off, 17% (17/101) of the myocarditis cases (31 [6, 85] days prior to ICI start) received the FV compared to 34% (69/201, *p* = 0.002 for rate comparison) of controls (44 [13, 58] days prior to ICI start, Table 1). A complete description comparing the myocarditis cases using the 3-month time-cut off stratified by FV status is presented in Additional file 1: Table S1. We additionally used a third cut-off time to define FV status. In this third cut-off, we defined FV as only those who were administered the FV while on ICI. When FV status was restricted to those administered the FV while on ICI, the rates of FV in myocarditis cases during the period while on ICI therapy was 8% (8/101) compared to 17% (34/201) of controls who did not develop myocarditis (*p* = 0.04, a complete description of comparisons using this final threshold is not shown). We also tested whether there was temporal pattern in myocarditis presentation. There was no difference found in the temporal pattern of presentation with myocarditis, with 31% occurring in Spring, 22% in Summer, 21% in Autumn and 26% in Winter (*p* = 0.31).

### Comparison within myocarditis cases of those that were and were not administered the FV

When myocarditis cases who received the FV in the 6 months prior to ICI were compared to myocarditis cases who did not receive the FV, there was no difference with respect to age (69 ± 8 vs. 66 ± 20 years, *p* = 0.60), sex (male, 68 vs. 74%, *p* = 0.58), or cardiovascular risk factors (smoking history 48 vs. 47%, *p* = 0.95; hypertension 58 vs. 60%, *p* = 0.42; diabetes mellitus 30 vs. 21%, *p* = 0.36, Table 3). There was also no difference in the use of monotherapy or combined ICI treatment, as well as overall ICIs used among myocarditis cases when stratified by vaccination status. A complete description of the comparisons of ICI therapies between myocarditis cases who were and were not administered the FV is presented in Table 3. The occurrence of other irAEs was compared within the myocarditis cases, and 36% of cases vaccinated compared to 55% of unvaccinated cases had further immune side effects during treatment (*p* = 0.10). Cases administered the vaccination were not at increased risk of other immune side effects during treatment (FV vs. no FV, hypophysitis 4 vs. 7%, *p* = 1.00; hepatitis 4 vs. 9%, *p* = 0.68; colitis 8 vs. 9%, *p* = 1.00; dermatitis 0 vs. 8%, *p* = 0.33; neurological 4 vs. 13%, *p* = 0.28 or gastritis 0 vs. 4%, *p* = 0.57 (Table 3)). In contrast, myocarditis cases administered the FV were less likely to have prior ICI-related pneumonitis (12 vs. 36%, *p* = 0.03) (Table 3). When stratifying the groups by FV status, there was no difference in the LVEF (46 ± 15 vs. 50 ± 16%, *p* = 0.28, Table 3) but serum troponin, a measure of myocardial injury, was higher among cases who did not receive the FV. Specifically, when compared to unvaccinated cases, cases administered the FV had a 3-fold lower troponin T level (0.12 [0.02, 0.47] vs. 0.40 [0.11, 1.26] ng/ml, *p* = 0.02) (Table 3).

**Table 3:** Comparison of Myocarditis cases with and without Flu vaccination (FV)

	FV (*n* = 25)	No FV (*n* = 76)	*P Value*
Age at start of ICI, yrs	69 ± 8	66 ± 20	0.60
Male	17 (68)	56 (74)	0.58
*CV risk factors*			
Current or prior smoking	10 (48)	30 (47)	0.95
Hypertension	14 (58)	45 (60)	0.89
Diabetes mellitus	7 (30)	15 (21)	0.36
No CV risk factors	4 (16)	19 (25)	0.35
Coronary artery disease	3 (15)	9 (13)	0.73
Stroke	1 (5)	6 (8)	1.00
Heart failure	1 (5)	4 (6)	1.00
COPD	5 (28)	7 (10)	0.12
Obstructive sleep apnea	0	6 (9)	0.60
Chronic kidney disease^a^	2 (11)	7 (10)	1.00
Body mass index, kg/m^2^	28 ± 5	28 ± 7	0.90
*Primary cancer type*			
Head and neck	0	5 (7)	0.33
Hodgkin’s lymphoma	0	2 (3)	1.00
Melanoma	12 (48)	32 (42)	0.61
Lung cancer	6 (24)	11 (14)	0.35
Pancreatic	2 (8)	0	0.06
Renal cell carcinoma	2 (8)	4 (5)	0.64
Glioblastoma	0	2 (3)	1.00
Other	3 (12)	20 (26)	0.18
*Prior chemotherapy or radiation*			
Radiation	4 (16)	25 (33)	0.11
Anthracyclines	1 (4)	5 (7)	1.00
Cyclophosphamide	1 (4)	1 (1)	0.44
Gemcitabine	2 (8)	3 (4)	0.60
Taxanes	2 (8)	4 (5)	0.64
Carboplatin	2 (8)	6 (8)	1.00
VEGF Inhibitors	0	1 (1)	1.00
*Single agent* vs. *combined ICI*			
Combination	8 (32)	20 (26)	0.61
Monotherapy	17 (68)	56 (74)	0.61
*Combined ICI*			
Ipilimumab + nivolumab	8 (32)	16 (21)	0.27
Ipilimumab + pembrolizumab	0	1 (1)	1.00
Tremelimumab + avelumab	0	1 (1)	1.00
Tremelimumab + durvalumab	0	2 (3)	1.00
*Monotherapy ICI* ^b^			
Pembrolizumab (anti-PD1)	7 (28)	28 (37)	0.42
Nivolumab (anti-PD1)	7 (28)	18 (24)	0.66
Ipilimumab (anti-CTLA4)	2 (8)	4 (5)	0.64
Tremelimumab (anti-CTLA4)	1 (4)	0	0.25
Atezolizumab (anti-PDL1)	0	6 (8)	0.33
Avelumab (anti-PDL1)	0	0	1.00
Durvalumab (anti-PDL1)	0	0	1.00
*Overall types of ICI*			
Any anti-PD1	22 (88)	63 (83)	0.75
Any anti-CTLA4	11 (44)	24 (32)	0.33
Any anti-PDL1	0	9 (12)	0.11
Days of follow-up [IQR]	223 [111, 324]	162 [86, 364]	0.32
*Other immune side effects during treatment* ^c^			
No other immune side effects	16 (64)	34 (45)	0.10
Hypophysitis/pituitary/adrenal	1 (4)	5 (7)	1.00
Pneumonitis	3 (12)	26 (36)	0.03
Hepatitis	1 (4)	7 (9)	0.68
Colitis	2 (8)	7 (9)	1.00
Dermatitis	0	6 (8)	0.33
Neurological	1 (4)	10 (13)	0.28
Gastritis	0 (0)	3 (4)	0.57
*Myocarditis presentation* ^c^			
Chest pain	15 (60)	50 (66)	0.60
Shortness of breath	6 (25)	21 (28)	0.75
Orthopnea	6 (26)	18 (24)	0.86
Paroxysmal nocturnal dyspnea	2 (9)	7 (9)	1.00
Fatigue	8 (40)	27 (46)	0.65
*Admission examination*			
Jugular venous distension	8 (32)	24 (32)	1.00
Crackles on lung exam	8 (32)	36 (47)	0.25
*Admission vitals*			
Heart rate, beats/min	92 ± 16	89 ± 24	0.67
Systolic blood pressure, mmHg	126 ± 17	126 ± 21	0.89
Diastolic blood pressure, mmHg	70 ± 10	72 ± 11	0.48
Respiratory rate, rate, min	19 ± 2	22 ± 14	0.38
*Oxygen requirement and delivery* ^d^			
Room air	13 (72)	51 (75)	0.61
Nasal cannula	5 (28)	13 (19)	0.61
Intubated	0	4 (6)	0.61
*Echocardiography, myocarditis admission*			
LVEF^e^, %	46 ± 15	50 ± 16	0.28
LVIDD, mm	45 ± 11	48 ± 6	0.15
*Admission cardiac enzymes*			
Troponin T, ng/ml	0.12 [0.02,0.47]	0.40 [0.11,1.26]	0.02
BNP or NT-pro BNP, pg/ml	568 [421,987]	600 [215,4275]	0.82
*Outcomes: MACE* ^f^			
Cumulative MACE	6 (24)	45 (59)	0.002
Complete heart block	2 (9)	14 (19)	0.35
Cardiogenic shock	2 (9)	15 (20)	0.35
Cardiac arrest	2 (9)	13 (17)	0.51
Cardiovascular death	4 (36)	28 (72)	0.04

Values are mean ± SD or n (%), or median [interquartile range]. ^a^Chronic kidney disease = glomerular filtration rate < 60 ml/min/1.73 m^2^. ^b^If most recent ICI therapy was monotherapy. ^c^Can include more than 1. ^d^Of available cases (18 vaccinated, 69 unvaccinated).^e^All vaccinated cases [25] and 74 of the 76 unvaccinated cases had an admission echocardiogram. ^f^Cases may have had more than one MACE, but only first event encountered was included in analysis. *CV* cardiovascular, *ICI* immune checkpoint inhibitors, *anti-CTLA4* anti-cytotoxic T-lymphocyte-associated protein 4, *anti-PD1* anti-programmed cell death protein 1, *anti-PDL1* anti-programmed death-ligand 1, *LVEF* left ventricular ejection fraction, *LVIDD* left ventricular internal dimension diameter, *BNP* brain natriuretic peptide, *NT- pro BNP* N-terminal pro BNP

### Major adverse cardiac events

**Fig. 1:**
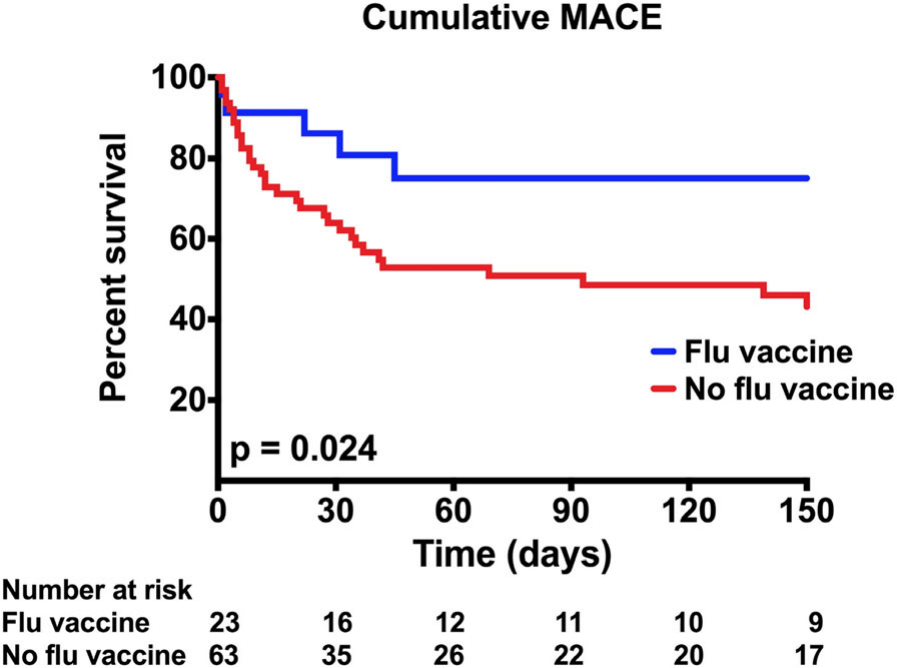
Kaplan-Meier curve showing cumulative MACE among cases stratified by flu vaccination status

The median follow-up of myocarditis cases was 175 days (interquartile range 89 to 363 days] (Table 2) and during this follow-up period, 47% (47/101) of all myocarditis cases experienced a MACE: CHB (*n* = 16), cardiogenic shock (*n* = 17), cardiac arrest (*n* = 15), or cardiovascular death (*n* = 32, Table 3). Myocarditis cases who received the FV were at a lower risk of cumulative MACE when compared to unvaccinated cases (cumulative MACE 24 vs. 59%, *p* = 0.002) (Fig. 1). When the individual components of MACE were compared, vaccinated cases were less likely to have a cardiovascular death when compared to cases not administered the flu vaccine (36 vs. 72%, *p* = 0.04, Table 3). The rates of the other individual components were non-significantly lower among those administered the FV: complete heart block (9 vs. 19%, FV vs. no FV, *p* = 0.35), cardiogenic shock (9 vs. 20%, *p* = 0.35), or cardiac arrest (9 vs. 17%, *p* = 0.51, Table 3).

## Discussion

We tested the association between FV and the development of myocarditis among patients on ICIs, and the effect of FV status on presentation and outcomes among patients who develop ICI-myocarditis. In our retrospective study of 101 ICI-related myocarditis cases, we found that 25% were vaccinated against influenza. In comparison, rate of vaccination for influenza was higher (40%) among 201 control patients on ICIs who did not develop myocarditis. This first analysis included those administered the FV from 6 months prior to commencing ICI therapy or during therapy. Similar findings of lower rates of vaccination against flu among myocarditis cases were noted when using a 3-month cut-off or, when restricting FV to administration during ICI therapy only and restricting to cases from the same institution from where the controls were derived. Among myocarditis cases, serum troponin, a measure of myocardial injury used to detect myocarditis, was 3-fold higher among myocarditis cases not administered the FV. This increase in serum troponin among unvaccinated myocarditis cases was associated with an increase in subsequent major adverse cardiac events. Specifically, during follow up, the rate of cumulative MACE among unvaccinated cases was more than double the adverse event rate seen among myocarditis cases administered the FV. Additional parallel findings of importance related to pneumonitis were noted that were not the primary focus of this paper. As compared to controls who did not develop myocarditis, the rates of pneumonitis were higher among myocarditis cases; and in analyses restricted to myocarditis cases, the rates of pneumonitis were higher among those cases not administered the FV.

Data testing the association between FV status and immune-mediated adverse events among patients on ICI therapy have provided conflicting results. In a single center study among patients with lung cancer, influenza vaccination during treatment with anti-PD1 induced an adequate serological protection from influenza, an increased inflammatory response and heightened the risk for immune-related adverse events [27]. There are differences in our study and that prior study that may explain the discordant findings. Specifically, we included patients with all types of cancers who were on an ICI, we included all types of ICI therapies and we only primarily focused on one type of adverse event, myocarditis. We focused on myocarditis for the following four reasons: 1) myocarditis is an uncommon but serious complication of ICI therapy, 2) myocarditis can occur among patients with active influenza infection, 3) vaccination against influenza has been associated with a reduction in cardiovascular events in broad populations [40, 41], 4) the risk for cardiovascular events is increased broadly among patients with cancer, Data among broad populations showing a beneficial effect of FV on cardiovascular events are robust. For example, in a large meta-analysis of randomized controlled trials of nearly 7000 patients, the administration of FV was associated with a lower risk of MACE [30]. Other studies have also suggested that the use of the FV vaccine may be safe among patients on an ICI. Specifically, a recent single center study suggested that the seasonal influenza vaccination is safe and may be beneficial for patients on ICI with reduced rates of hospital admissions from flu-related and immunotherapy-related adverse events [29]. This latter study also included patients with all types of cancer and ICI therapies.

In supportive findings of a protective effect of FV among patients on an ICI, we found that biomarkers of risk in general myocarditis and for adverse events among patients who develop ICI myocarditis were higher among unvaccinated cases. We previously noted that serum troponin, a sensitive marker for myocardial injury is elevated among most patients with ICI myocarditis and the degree of elevation of serum troponin is a predictor of adverse cardiovascular events among patients who develop myocarditis on an ICI [15]. In this current study, troponin levels were higher among unvaccinated cases with ICI myocarditis compared to cases with myocarditis who were administered the FV. We also noted that serious adverse cardiac events were increased among patients who developed ICI myocarditis and were not previously administered the FV.

Although not the primary focus of this paper our findings regarding other immune-related adverse events, specifically pneumonitis, merit discussion. Our rates of any grade of other irAEs were 36 and 55% in the vaccinated and unvaccinated cases, respectively. These are comparable to the 37% irAEs in the vaccinated group and 43% in the unvaccinated group reported in the discussed single center study showing a protective effect of FV [29]. However, these rates are still lower than the rate of 52.2% of previously vaccinated patients developing any grade irAEs in the study of Läubli and colleagues [27]. We found higher rates of pneumonitis in the population without FV, Pneumonitis and pneumonia are important causes of influenza-associated morbidity and mortality among broad populations [42, 43] and FV has been shown to reduce morbidity and mortality among at-risk individuals [40, 43], including patients with cancer [44–46]. In our cohort, vaccinated cases had lower rates of immune-related pneumonitis compared to unvaccinated cases. This may be explained by the protective nature of the FV against pneumonitis and pneumonia and support the need for prospective randomized studies in this at-risk population.

This study has some limitations that merit discussion. This is the largest registry of patients with ICI-myocarditis; however, this was a retrospective case-control study where cases were derived from multiple institutions and controls were derived from a single institution. To address this, we also compared the rates of FV within cases and controls from the same institution and found similar results of a lower rate of FV among patients on an ICI who got myocarditis. Additionally, as this was a retrospective study, the type of influenza vaccination, the specific antibody titers and measures of inflammatory response were not recorded. Also, the choice of whether patients were administered the FV was at the discretion of the clinician involved in their care, which differed between the centers but also locally within each center. Ideally to test the association of the FV and ICI myocarditis, a prospective study comparing all ICI patients with and without FV who develop myocarditis, or a randomized clinical trial would be warranted. However, with a low incidence rate of ICI-myocarditis (~ 0.5–1.0% or less) [14, 15, 20, 21], to test this association adequately, a large cohort of subjects would be required. In addition, there are currently no available systematic screening approaches for myocarditis among patients on ICIs and diagnosis is based on physician’s suspicion. Therefore, a prospective approach may also lead to an underestimation in the incidence of myocarditis and the effect of the FV. Finally, this study does not provide a mechanism by which the FV may be protective. Indeed, the mechanism underlying the protective effect of the FV against cardiovascular events in the general population is also unclear [30], but potential mechanisms include rupture of a vulnerable atherosclerotic plaque, heart failure, or, relevant to this study, myocarditis [32, 47–49]. Direct involvement of influenza in the myocardium, leading to myocarditis, is uncommon with rates of up to 10% reported depending on methods of detection used [31] and influenza infection can cause myocarditis by direct cytolysis of the myocyte causing necrosis, but also the host immune response to the virus may play an important role [50].

## Conclusion

In summary, the administration of the FV was not associated with an increased risk of subsequent myocarditis among patients on ICI. In contrast, rates of influenza vaccination were lower among patients who did develop myocarditis on ICI, and the influenza vaccine was associated with a lower rate of ICI-related pneumonitis. At presentation, myocarditis cases administered the FV had lower troponin levels and, in follow-up, had lower rates of cumulative MACE. There is a clear need to establish the safety status of influenza vaccination among cancer patients treated with ICIs, as our data suggest that it may be protective. Further large studies are warranted to test and validate these important findings.

## Additional file

Additional file 1: **Table S1.** (3-month cut-off): Comparison of Myocarditis cases with^‡^ and without Flu vaccination (FV). (DOCX 22 kb)
