# Supplementary material for: Influenza vaccination and myocarditis among patients receiving immune checkpoint inhibitors
Source: J Immunother Cancer. 2019 Feb 22;7:53. doi: 10.1186/s40425-019-0535-y (PMC6387531; doi:10.1186/s40425-019-0535-y)
Supplement: Supplementary file 1 — Table S1. (3-month cut-off): Comparison of Myocarditis cases with‡ and without Flu vaccination (FV). (DOCX 22 kb) [file 40425_2019_535_MOESM1_ESM.docx]

| **Additional file 1: Table S1. (3-month cut-off): Comparison of Myocarditis cases with**^‡^  **and without Flu vaccination (FV)** | | | | | |
| --- | --- | --- | --- | --- | --- |
|  | | | FV  (n=17) | No FV  (n=84) | *P Value* |
|  |  | | |  |  |
| Age at start of ICI, yrs | | 70±7 | | 66±19 | 0.39 |
| Male | | 12 (71) | | 61 (73) | 1.00 |
| *CV risk factors* | |  | |  |  |
| Current or prior smoking | | 8 (53) | | 32 (46) | 0.59 |
| Hypertension | | 11 (65) | | 48 (59) | 0.64 |
| Diabetes mellitus | | 6 (38) | | 16 (21) | 0.19 |
| No CV risk factors | | 2 (12) | | 21 (25) | 0.35 |
| Coronary artery disease | | 2 (13) | | 10 (13) | 1.00 |
| Stroke | | 0 | | 7 (9) | 0.60 |
| Heart failure | | 1 (7) | | 4 (5) | 1.00 |
| COPD | | 5 (36) | | 7 (10) | 0.02 |
| Obstructive sleep apnea | | 0 | | 6 (8) | 0.59 |
| Chronic kidney disease* | | 2 (14) | | 7 (10) | 0.64 |
| Body mass index, kg/m^2^ | | 28±5 | | 28±7 | 0.80 |
| *Primary cancer type* | |  | |  |  |
| Head and neck | | 0 | | 5 (6) | 0.59 |
| Hodgkin’s lymphoma | | 0 | | 2 (2) | 1.00 |
| Melanoma | | 9 (53) | | 35 (42) | 0.39 |
| Lung cancer | | 5 (29) | | 12 (14) | 0.16 |
| Pancreatic | | 1 (6) | | 1 (1) | 0.31 |
| Renal cell carcinoma | | 1 (6) | | 5 (6) | 1.00 |
| Glioblastoma | | 0 | | 2 (2) | 1.00 |
| Other | | 1 (6) | | 22 (26) | 0.11 |
| *Prior chemotherapy or radiation* | |  | |  |  |
| Radiation | | 3 (18) | | 26 (31) | 0.38 |
| Anthracyclines | | 1 (6) | | 5 (6) | 1.00 |
| Cyclophosphamide | | 1 (6) | | 1 (1) | 0.31 |
| Gemcitabine | | 1 (6) | | 4 (5) | 1.00 |
| Taxols | | 2 (12) | | 4 (5) | 0.27 |
| Carboplatin | | 2 (12) | | 6 (7) | 0.62 |
| VEGF Inhibitors | | 0 | | 1 (1) | 1.00 |
| *Single agent vs. combined ICI* | |  | |  |  |
| Combination | | 6 (35) | | 22 (26) | 0.55 |
| Monotherapy | | 11 (65) | | 62 (74) | 0.55 |
| *Combined ICI* | |  | |  |  |
| Ipilimumab + nivolumab | | 6 (35) | | 18 (21) | 0.23 |
| Ipilimumab + pembrolizumab | | 0 | | 1 (1) | 1.00 |
| Tremelimumab + avelumab | | 0 | | 1 (1) | 1.00 |
| Tremelimumab + durvalumab | | 0 | | 2 (2) | 1.00 |
| *Monotherapy ICI*† | |  | |  |  |
| Pembrolizumab (anti-PD1) | | 4 (24) | | 31 (37) | 0.29 |
| Nivolumab (anti-PD1) | | 5 (29) | | 20 (24) | 0.76 |
| Ipilimumab (anti-CTLA4) | | 1 (6) | | 5 (6) | 1.00 |
| Tremelimumab (anti-CTLA4) | | 1 (6) | | 0 | 0.17 |
| Atezolizumab (anti-PDL1) | | 0 | | 6 (7) | 0.59 |
| Avelumab (anti-PDL1) | | 0 | | 0 | 1.00 |
| Durvalumab (anti-PDL1) | | 0 | | 0 | 1.00 |
| *Overall types of ICI* | |  | |  |  |
| Any anti-PD1 | | 15 (88) | | 70 (83) | 1.00 |
| Any anti-CTLA4 | | 8 (47) | | 27 (32) | 0.27 |
| Any anti-PDL1 | | 0 | | 9 (11) | 0.35 |
|  | |  | |  |  |
| Days of follow-up [IQR] | | 223 [88, 324] | | 167 [92, 364] | 0.53 |
| *Other immune side effects during*  *Treatment*** | |  | |  |  |
| No other immune side effects | | 11 (65) | | 39 (46) | 0.17 |
| Hypophysitis/pituitary/adrenal | | 1 (6) | | 5 (6) | 1.00 |
| Pneumonitis | | 2 (12) | | 28 (33) | 0.08 |
| Hepatitis | | 0 | | 8 (10) | 0.35 |
| Colitis | | 2 (12) | | 7 (8) | 0.65 |
| Dermatitis | | 0 | | 6 (7) | 0.59 |
| Neurological | | 0 | | 11 (13) | 0.20 |
| Gastritis | | 0 | | 3 (4) | 1.00 |
|  | |  | |  |  |
| *Myocarditis presentation*** | |  | |  |  |
| Chest pain | | 11 (65) | | 54 (64) | 0.97 |
| Shortness of breath | | 3 (19) | | 24 (29) | 0.55 |
| Orthopnea | | 3 (20) | | 21 (26) | 0.76 |
| Paroxysmal nocturnal dyspnea | | 2 (13) | | 7 (8) | 0.62 |
| Fatigue | | 7 (58) | | 28 (42) | 0.29 |
| *Admission examination* | |  | |  |  |
| Jugular venous distension | | 5 (33) | | 27 (35) | 0.92 |
| Crackles on lung exam | | 6 (43) | | 38 (46) | 0.81 |
| *Admission vitals* | |  | |  |  |
| Heart rate, beats/min | | 94±17 | | 89±23 | 0.39 |
| Systolic blood pressure, mmHg | | 130±18 | | 125±21 | 0.45 |
| Diastolic blood pressure, mmHg | | 70±11 | | 71±11 | 0.78 |
| Respiratory rate, rate, min | | 19±3 | | 21±13 | 0.62 |
| *Oxygen requirement and delivery**** | |  | |  |  |
| Room air | | 8 (62) | | 56 (77) | 0.23 |
| Nasal cannula | | 5 (38) | | 13 (18) | 0.23 |
| Intubated | | 0 | | 4 (5) | 0.23 |
| *Echocardiography, myocarditis admission* | |  | |  |  |
| New LVEF*¶*, % | | 43±14 | | 50±16 | 0.07 |
| Change in LVEF from baseline, % | | 22±12 | | 13±14 | 0.08 |
| LVIDD, mm | | 45±13 | | 48±6 | 0.21 |
| *Admission cardiac enzymes* | |  | |  |  |
| Troponin T, ng/ml [IQR] | | 0.15 [0.02, 0.45] | | 0.40 [0.08, 1.00] | 0.11 |
| BNP or NT-pro BNP, pg/ml [IQR] | | 568 [361, 1179] | | 600 [218, 4224] | 0.89 |
| *Outcomes: MACE*†† | |  | |  |  |
| Cumulative MACE | | 5 (29) | | 46 (55) | 0.06 |
| Complete heart block | | 2 (12) | | 14 (17) | 0.73 |
| Cardiogenic shock | | 2 (12) | | 15 (19) | 0.73 |
| Cardiac arrest | | 1 (6) | | 14 (17) | 0.45 |
| Cardiovascular death | | 3 (33) | | 29 (71) | 0.06 |
| Values are mean ± SD or n (%), or median [interquartile range]. ‡Flu vaccination restricted to within 3 months prior to commencing ICI or whilst on ICI therapy. *Chronic kidney disease = glomerular filtration rate <60 ml/min/1.73 m^2^. †If most recent ICI therapy was monotherapy. **Can include more than 1. ***Of available cases (13 vaccinated, 74 unvaccinated). ¶Eighty two out of the 84 unvaccinated cases and all vaccinated cases (17) had an admission echocardiogram. ††Cases may have had more than one MACE, but only first event encountered was included in analysis. CV = cardiovascular; ICI = immune checkpoint inhibitors; anti-CTLA4 = anti-cytotoxic T-lymphocyte-associated protein 4; anti-PD1 = anti-programmed cell death protein 1; anti-PDL1 = anti-programmed death-ligand 1; LVEF = left ventricular ejection fraction; LVIDD = left ventricular internal dimension diameter; BNP = brain natriuretic peptide; NT- pro BNP = N-terminal pro BNP. | | | | | |
